# The Impact of Bullying Victimization on School Adjustment: A Moderated Mediation Model

**DOI:** 10.3390/bs16091590

**Published:** 2026-09-07

**Authors:** Hanwen Chen, Yiyang Wang, Tianci Lu, Qingcan Zhou, Hao Chen, Qingyu Liang, Chen Zhang, Jun Yan

**Affiliations:** College of Physical Education, Yangzhou University, Yangzhou 225000, China; dx120230091@stu.yzu.edu.cn (H.C.); mz120250786@stu.yzu.edu.cn (Y.W.); dx120240094@stu.yzu.edu.cn (T.L.); dx120250103@stu.yzu.edu.cn (Q.Z.); dx120250102@stu.yzu.edu.cn (H.C.); mz120240821@stu.yzu.edu.cn (Q.L.); mz120240831@stu.yzu.edu.cn (C.Z.)

**Keywords:** physical activity, bullying victimization, psychological resilience, school adjustment, junior high school students

## Abstract

Bullying victimization is a major risk factor for adolescents’ school adjustment, yet the mechanisms linking these experiences remain unclear. This study examined whether psychological resilience mediates the association between bullying victimization and school adjustment among junior high school students, and whether physical activity moderates this process. Using a cross-sectional design, data were obtained from 19,149 junior high school students in the Adolescent Health Theme Database. Regression, Bootstrap mediation, and moderated mediation analyses were conducted. Bullying victimization significantly and negatively associated with school adjustment (*β* = −0.460, *p* < 0.001). Psychological resilience partially accounted for this association: bullying victimization was negatively associated with psychological resilience (*β* = −0.314, *p* < 0.001), and psychological resilience was positively associated with school adjustment (*β* = 0.472, *p* < 0.001). The indirect effect was −0.149, with a 95% confidence interval of −0.157 to −0.140, accounting for 32.391% of the total effect. Physical activity moderated both the direct association between bullying victimization and school adjustment (*β* = 0.055, *p* < 0.001) and the association between psychological resilience and school adjustment (*β* = 0.020, *p* < 0.001). These findings indicate that bullying victimization is negatively associated with school adjustment, both directly and indirectly through its association with psychological resilience. Physical activity may serve as a modest but statistically significant protective factor by attenuating the adverse association between bullying victimization and school adjustment and strengthening the positive association between psychological resilience and school adjustment.

## 1. Introduction

Adolescence is a critical period characterized by rapid physical and psychological development, increasing self-awareness, and growing demands for academic and social adaptation (Steinberg, 2014; Sawyer et al., 2018). During this stage, adolescents need to adapt to school norms, establish peer relationships, and complete academic tasks. As a central context for adolescents’ daily life and social development, school not only serves the function of knowledge transmission but also influences their emotional development, interpersonal interactions, and mental health (Eccles & Roeser, 2011). Therefore, school adjustment can be understood as a multidimensional construct that reflects students’ academic, interpersonal, emotional, and behavioral adaptation to the school environment, including learning engagement, school integration, teacher–student relationships, peer relationships, and sense of school belonging (Azpiazu Izaguirre et al., 2024). Correspondingly, poor school adjustment is not simply reflected in declining academic achievement or behavioral deviance. Instead, it involves multidimensional psychosocial adjustment difficulties, such as aggression, oppositional defiance, social withdrawal, peer rejection, and school burnout, and is closely associated with academic performance, behavioral problems, and emotional health (Azpiazu et al., 2025). Junior high school students face multiple changes, including physical and psychological development, increased academic demands, and the reconstruction of interpersonal relationships. In particular, during the transition from primary school to secondary school, increased academic demands, intensified competition, reduced perceived teacher support, and decreased classroom belonging may pose challenges to students’ academic and emotional adjustment (Engels et al., 2019). More broadly, this study contributes to ongoing debates on how adolescents maintain school adjustment under peer-related adversity. In particular, it responds to the question of whether school adjustment in the context of bullying victimization is primarily shaped by risk exposure itself or by the interplay between risk factors and protective resources. From this perspective, examining both risk and protection can provide a more balanced understanding of adolescents’ adjustment in school contexts.

Therefore, exploring the influencing factors and underlying mechanisms of school adjustment among junior high school students can help identify risk sources and protective factors, thereby providing theoretical evidence and practical implications for improving school adjustment and promoting healthy physical and psychological development.

Although previous studies have confirmed the close association between bullying victimization and adolescents’ school adjustment and have revealed the important role of psychological resilience in the effects of negative experiences on individual development, the internal psychological mechanisms and boundary conditions underlying this relationship remain insufficiently examined. Existing research has mainly focused on negative psychological outcomes, such as anxiety, depression, and loneliness, while relatively less attention has been paid to school adjustment, which reflects adolescents’ overall integration into school life. Meanwhile, although physical activity may buffer the negative associations between bullying victimization and school adjustment, few studies have incorporated physical activity into the pathways linking bullying victimization with school adjustment. It remains unclear whether physical activity modifies the direct and indirect pathways through which bullying victimization affects school adjustment. Psychological resilience and physical activity were selected because they represent two complementary protective resources. Psychological resilience reflects an internal coping resource that may explain how bullying victimization is translated into school adjustment outcomes. Physical activity, in contrast, represents a modifiable behavioral resource that can be strengthened through school-based programs and daily educational practice. Examining these two factors together allows this study to consider both the psychological mechanism and the behavioral condition under which bullying victimization affects school adjustment. Therefore, based on stress-coping theory (Lazarus & Folkman, 1984) and protective factor theory (Fergus & Zimmerman, 2005; Zimmerman, 2013), this study constructed a moderated mediation model among junior high school students to examine the mediating role of psychological resilience and the moderating role of physical activity. To make the research focus explicit, the present study examined two central questions: first, whether psychological resilience explains the association between bullying victimization and school adjustment; and second, whether physical activity changes the strength of the direct and indirect pathways linking bullying victimization to school adjustment. By doing so, this study aimed to clarify the psychological pathways underlying the association between bullying victimization and school adjustment among junior high school students and to provide reasonable and effective recommendations for improving their school adjustment.

### 1.1. Literature Review

#### 1.1.1. School Adjustment and Bullying Victimization

Previous studies have shown that adolescents’ school adjustment is influenced by multiple factors. External support systems, such as family, school, and peers, as well as internal psychological resources, such as psychological resilience and positive emotions, may all affect school adjustment (Azpiazu Izaguirre et al., 2024; Chan et al., 2022). Poor school adjustment is a relatively common developmental difficulty during adolescence. In China, approximately 18–35% of adolescents experience problems related to poor school adjustment (Yao et al., 2025). Studies conducted in other countries have also found that 5.9% of children and adolescents reach clinically significant levels of poor school adjustment, while another 16.2% are at risk (Bernaras et al., 2017). Among these factors, negative experiences in peer relationships, especially bullying victimization, may weaken adolescents’ sense of school belonging and academic engagement, further influencing their academic development (L. Li et al., 2020). Bullying victimization generally refers to repeated, or potentially repeated, intentional physical, verbal, or relational harm inflicted on adolescents within peer relationships characterized by an imbalance of power (Smith, 2016; Merrin et al., 2024). Existing international evidence indicates that bullying is not a local or occasional problem but a widespread global phenomenon among children and adolescents. For example, the latest publicly available data from the United Nations system indicate that bullying is a widespread global issue (UNESCO, 2026), and the 2021/2022 Health Behaviour in School-aged Children survey released by the WHO Regional Office for Europe showed that approximately 11% of adolescents reported being bullied at school, while 15% reported experiencing cyberbullying across 44 countries and regions in Europe, Central Asia, and Canada (World Health Organization Regional Office for Europe, 2024).

Studies have found that students who experience peer bullying show higher levels of psychological distress, depression, and anxiety than those who have not been bullied (Ajibewa et al., 2025). Such psychological distress may undermine their resources for coping with learning tasks and school-based interpersonal relationships, thereby reducing their adaptive capacity in terms of academic performance, peer relationships, and emotional engagement (Burger, 2022). Taken together, prior studies have established bullying victimization as an important risk factor for adolescents’ maladjustment. However, much of this literature has emphasized negative psychological or behavioral outcomes, whereas school adjustment as a multidimensional indicator of adolescents’ integration into school life has received relatively less attention. Moreover, existing studies have not sufficiently clarified the psychological pathway through which bullying victimization is linked to school adjustment.

#### 1.1.2. Psychological Resilience

Psychological resilience refers to individuals’ ability to maintain positive adjustment and development in the face of adversity or major challenges, and it is an important psychological resource for coping with social stress and a key indicator of mental health (Masten, 2018). As an internal protective factor, psychological resilience can help individuals overcome the negative effects of multiple risk factors (Sisto et al., 2019). In contrast, students with higher levels of psychological resilience are better able to maintain emotional stability when facing academic pressure, interpersonal conflict, and negative evaluation, and are more likely to adopt positive problem-solving and cognitive regulation strategies and remain engaged in school life (Azpiazu Izaguirre et al., 2024). Previous studies have also shown that psychological resilience mediates the associations between bullying victimization and negative outcomes such as anxiety, depression, and self-injury (Fang et al., 2022). These studies highlight the protective role of psychological resilience, but they have mainly examined resilience in relation to mental health outcomes such as anxiety, depression, or self-injury. Less is known about whether psychological resilience explains the association between bullying victimization and school adjustment. Therefore, the present study extends previous work by positioning psychological resilience as a mechanism linking peer-related adversity to school-based adjustment.

#### 1.1.3. Physical Activity

As a modifiable and intervenable health behavior, physical activity can be continuously improved and consolidated through training, supportive environments, and sustained practice (van Sluijs et al., 2021; Kolovelonis et al., 2024). A large body of research has confirmed that regular physical activity helps alleviate depression, anxiety, and stress among adolescents and promotes the development of self-esteem, self-efficacy, and social adjustment (Biddle & Asare, 2011; Rodriguez-Ayllon et al., 2019). Existing studies have shown that physical activity is closely associated with adolescents’ school adjustment. A study of Chinese junior high school students found that physical activity positively predicted school adjustment, and that psychological resilience and coping style played a chain-mediating role in this relationship (Wu et al., 2024). Another study of high school students showed that physical activity was significantly associated with school adjustment, and that psychological resilience and self-control played a chain-mediating role in the effect of physical activity on school adjustment (H. Chen et al., 2024). These findings suggest that physical activity may not only directly promote adolescents’ school adjustment but may also support their adaptation to the school environment by enhancing psychological resilience, self-regulation, and positive coping resources. Although these findings support the positive role of physical activity, most existing studies have treated physical activity as a direct predictor of adjustment. Less attention has been paid to whether physical activity changes the strength of the association between bullying victimization and school adjustment or whether it enhances the beneficial role of psychological resilience. Addressing this gap can clarify whether physical activity functions not only as a promotive factor but also as a protective condition in the context of bullying victimization.

### 1.2. Theoretical Framework and Hypothesis Development

The present study draws on stress-coping theory and protective factor theory because these frameworks correspond directly to the core components of the proposed model. Stress-coping theory explains how stressful experiences, such as bullying victimization, may influence adjustment outcomes through individuals’ appraisal and coping resources. Protective factor theory further explains why some adolescents may maintain better adjustment despite exposure to risk, emphasizing the role of internal and external protective resources. Therefore, stress-coping theory provides the logic for the mediating role of psychological resilience, whereas protective factor theory provides the rationale for the moderating role of physical activity.

#### 1.2.1. Stress-Coping Theory and Bullying Victimization

According to stress-coping theory, individuals’ responses to external events are not directly determined by the events themselves but are formed through a dynamic process involving “stressful events–cognitive appraisal–coping responses–adjustment outcomes.” When an event is perceived as highly threatening and coping resources are insufficient, individuals are more likely to experience persistent psychological stress, which may further lead to negative emotions, behavioral withdrawal, and adjustment imbalance (Lazarus & Folkman, 1984). Because bullying victimization is characterized by intentional harm, repetition, and power imbalance (Smith, 2016), it may be regarded as a form of peer stress exposure with persistent threat implications rather than a one-time negative interpersonal event. Repeated experiences of ridicule, exclusion, aggression, or humiliation may extend adolescents’ perceived stress from specific bullying incidents to school life as a whole (Wolke & Lereya, 2015). Consistent with this view, adolescents who experience bullying victimization tend to report lower levels of school belonging and academic engagement, which in turn affects their academic performance and school adjustment (L. Li et al., 2020). Therefore, from the perspective of stress-coping theory, more severe bullying victimization may be associated with poorer school adjustment. Accordingly, this study proposes

**Hypothesis H1.** 
*Bullying victimization significantly and negatively predicts school adjustment*.

#### 1.2.2. Psychological Resilience as a Mediating Mechanism

Stress-coping theory further suggests that individuals do not passively endure the effects of stress; rather, coping resources play a key role in the process through which stress influences adjustment outcomes. Whether stressful events ultimately lead to maladjustment depends not only on the nature and intensity of the events themselves but also on individuals’ appraisal of their coping resources and the internal resources they are able to mobilize (Lazarus & Folkman, 1984). Adolescents who are chronically exposed to exclusion, aggression, and humiliation may develop feelings of helplessness and low efficacy, and may evaluate their coping abilities less positively when facing new school-related challenges. Therefore, bullying victimization may deplete adolescents’ internal coping resources, making it more difficult for them to maintain stable and positive psychological regulation, which may be reflected in reduced psychological resilience (Wolke & Lereya, 2015; Fang et al., 2022). Moreover, adolescents with higher psychological resilience are more likely to maintain positive adjustment, effectively cope with academic and interpersonal challenges, and remain engaged in school life (Azpiazu Izaguirre et al., 2024). Thus, psychological resilience may serve as an important psychological pathway through which bullying victimization affects school adjustment. Accordingly, this study proposes

**Hypothesis H2.** 
*Psychological resilience mediates the relationship between bullying victimization and school adjustment*.

#### 1.2.3. Physical Activity as a Risk-Protective Factor

In addition, individual development is not entirely determined by risk factors. Positive developmental outcomes also depend on the joint effects of internal and external protective resources. The theoretical framework of resilience proposes three basic models: the compensatory model, the protective model, and the challenge model. The protective model can be further divided into the risk-protective factor model and the protective factor-protective factor model (Fergus & Zimmerman, 2005; Zimmerman, 2013). The risk-protective factor model emphasizes that protective factors interact with risk factors to weaken the negative effects of risk on adverse outcomes. Compared with a risk-oriented perspective that focuses solely on problems and impairment, this model places greater emphasis on adolescents’ positive resources, plasticity, and amenability to intervention. Therefore, for adolescents who experience bullying victimization, higher levels of physical activity may help them obtain more positive emotional experiences and behavioral resources (H. Chen et al., 2024), and may buffer, to some extent, the negative psychological effects associated with bullying victimization (Feng et al., 2024; Y. Chen et al., 2025). This, in turn, may reduce the risk of impaired school adjustment. Accordingly, this study proposes

**Hypothesis H3.** 
*Physical activity moderates the relationship between bullying victimization and school adjustment*.

#### 1.2.4. Physical Activity and Psychological Resilience as Joint Protective Factors

Furthermore, from the perspective of the protective factor-protective factor model, different protective factors do not necessarily operate independently. Instead, they may jointly promote positive adjustment outcomes through synergistic or enhancing effects (Fergus & Zimmerman, 2005; Zimmerman, 2013). Psychological resilience, as an internal protective factor, can help adolescents better cope with academic pressure, interpersonal conflict, and negative evaluation. Physical activity, as an external and intervenable behavioral protective factor, may support adolescents’ adjustment to school life through participation in physical activities, peer interaction, and positive experiences. Accordingly, psychological resilience and physical activity may interact in the process of promoting school adjustment, such that higher levels of physical activity may provide more behavioral and social contextual support for the functioning of psychological resilience, thereby strengthening the positive effect of psychological resilience on school adjustment. Accordingly, this study proposes

**Hypothesis H4.** 
*Physical activity moderates the relationship between psychological resilience and school adjustment*.

**Figure 1:**
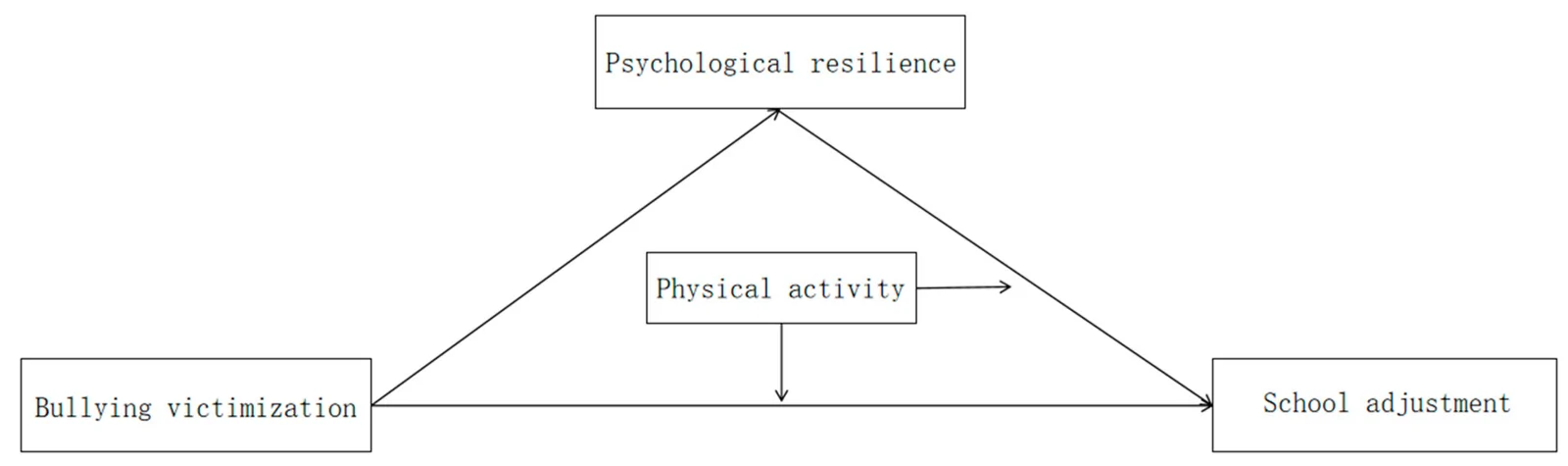
The hypothesized moderated mediation model.

Integrating these arguments, the present study proposes a moderated mediation model, as illustrated in Figure 1. This model was considered appropriate because it allows the study to examine both how bullying victimization is associated with school adjustment and when this association may be weaker or stronger. Bullying victimization is conceptualized as a peer-related stressor that may directly undermine school adjustment and indirectly reduce school adjustment by weakening psychological resilience. Physical activity is conceptualized as a modifiable behavioral protective factor that may buffer the direct negative association between bullying victimization and school adjustment and strengthen the positive association between psychological resilience and school adjustment. Thus, the hypothesized model links prior empirical findings with stress-coping theory and protective factor theory by simultaneously examining a risk pathway, a psychological mediating mechanism, and a behavioral moderating condition.

## 2. Materials and Methods

### 2.1. Data Source and Participants

The present study conducted a secondary analysis of de-identified data extracted from the Adolescent Health Theme Database 2024. No new survey data were collected for this study; instead, eligible cases and relevant variables were selected from the existing database for statistical analysis. The Adolescent Health Theme Database integrates adolescent health data from nationwide school district surveys conducted between 2019 and 2025, covering students in junior and senior high schools, and aims to establish a multidimensional and standardized adolescent health data system (Shandong University, 2021). Given that samples from Shandong Province comprised more than 80% of the total sample in the database, the present study focused on junior high school students from Shandong Province in 2024 as the study population. The original sample comprised 21,955 responses. After data screening and cleaning, invalid responses, including random responses, incomplete questionnaires, and questionnaires with excessive missing items, were removed. Finally, 19,149 valid responses were retained, yielding a valid-response retention rate of 87.2%. The final analytic sample included 10,161 boys (53.1%) and 8988 girls (46.9%), with a mean age of 14.68 years (SD = 1.078; range = 12–16 years).

### 2.2. Measures Available in the Database

#### 2.2.1. Chinese Version of the Physical Activity Questionnaire for Adolescents

In the source database, physical activity was represented by variables derived from the Chinese version of the Physical Activity Questionnaire for Adolescents. The original questionnaire was developed by Kowalski et al. (2004), and the Chinese version was revised and validated by X. Li et al. (2015). The questionnaire consists of nine items, of which the first eight items assess moderate-to-vigorous physical activity during the past week, including activity during physical education classes, lunchtime activity, after-school activity, evening activity, weekend activity, overall weekly activity, and overall daily activity. Each item is rated on a 5-point scale, with higher scores indicating higher levels of physical activity. In the analytic sample of the present study, *Cronbach’s α* for this scale was 0.935.

#### 2.2.2. School Social Behavior Scales, Second Edition

In the source database, school adjustment was assessed using 64 items derived from the School Social Behavior Scales, Second Edition (SSBS-2; Merrell, 2002). These items represent two conceptually related but distinguishable dimensions: Social Competence (Items 1–32) and Antisocial Behavior (Items 33–64). An example item is “being able to cooperate with other students most of the time.” Each item was rated on a 5-point Likert scale.

In accordance with the prespecified scoring instructions of the source database, an observed composite index of overall school adjustment was calculated by adding the Social Competence subtotal to the directionally transformed Antisocial Behavior subtotal: Σ(Items 1–32) + [160 − Σ(Items 33–64)]. No additional item-level reverse scoring was performed after this transformation. Possible composite scores ranged from 32 to 288, with higher scores indicating better overall school adjustment. This scoring procedure was retained to ensure consistency with the source database documentation and has also been used to operationalize overall school adjustment in previous applied research using the SSBS-2 (e.g., Y. Zhang et al., 2025; Yin et al., 2026).

Because the original SSBS-2 distinguishes Social Competence from Antisocial Behavior, we compared a single-factor model with a correlated two-factor model. The correlated two-factor model provided a better representation of the internal measurement structure. Importantly, the score used in the primary moderated-mediation analysis was not a factor score derived from the single-factor CFA model. Rather, it was the database-prespecified observed composite described above. Accordingly, this composite was interpreted as an operational index of overall school adjustment rather than as evidence that all 64 items formed a strictly unidimensional latent scale. The CFA results are reported in Supplementary Table S1. Cronbach’s α for the 64-item composite was 0.910.

#### 2.2.3. Assessment of Psychological Resilience

In the source database, psychological resilience was assessed using six items adapted from the Resilience Scale for Chinese Adolescents (RSCA; Hu & Gan, 2008). An example item is “In general, I have many things to be proud of.” Because the present study was a secondary analysis of an existing database, these six items were prespecified by the source database rather than selected by the present authors from the full RSCA. They constituted all psychological-resilience items available in the database, and all six items were retained in the present analysis without post hoc item selection or deletion. The database documentation described the six items as being adapted from the RSCA but did not designate them as belonging to a specific RSCA subscale or as constituting a separately validated short form. Therefore, the use of the six-item measure in the present study was based on the database-prespecified operationalization of psychological resilience rather than on an item-selection procedure conducted by the present authors.

Each item was rated on a 5-point Likert scale, with total scores ranging from 6 to 30. Higher scores indicated higher levels of psychological resilience. In the present analytic sample, the six-item measure demonstrated good internal consistency (Cronbach’s α = 0.885). Its one-factor structure was further evaluated using confirmatory factor analysis, with the standardized factor loadings and global model-fit indices reported in Supplementary Table S1.

#### 2.2.4. Assessment of Bullying Victimization

In the source database, bullying victimization was assessed using three items available in the Adolescent Health Theme Database 2024. These items reflected adolescents’ overall bullying victimization experiences in the Chinese school context, including experiences such as being looked down upon by teachers or people outside school. The measure assessed overall bullying victimization frequency rather than specific bullying subtypes (e.g., physical, verbal, relational, or cyber bullying). Responses were scored from 1 to 6, corresponding to “never,” “rarely,” “once every few months,” “at least once a month,” “once a week,” and “more than once a week.” Higher scores indicated more frequent bullying victimization. In the present analytic sample, *Cronbach’s α* for the three-item bullying victimization measure was 0.817.

### 2.3. Data Analysis

The present study analyzed secondary data extracted from the Adolescent Health Theme Database 2024. Statistical analyses were conducted using SPSS 26.0 and Mplus 8.3. First, descriptive statistics, including means, standard deviations, and percentages, were calculated to summarize participants’ sociodemographic characteristics and the main study variables. Second, confirmatory factor analyses (CFAs) were conducted using Mplus 8.3 to examine the construct validity of the multi-item psychological measures, including bullying victimization, psychological resilience, and school adjustment. Standardized factor loadings and model fit indices are presented in Supplementary Table S1. Third, Harman’s single-factor test was performed to assess potential common method bias (Podsakoff et al., 2003), and variance inflation factors were calculated to evaluate multicollinearity among variables. Fourth, Pearson correlation analyses were conducted to examine the bivariate associations among the study variables. Before mediation and moderated mediation analyses, the main continuous variables were standardized because they were measured on different scales. Standardization improved the comparability of regression coefficients and reduced potential multicollinearity when testing interaction effects.

Finally, Hayes’ PROCESS macro for SPSS, version 4.1, Models 4 and 15 were used to test the mediation and moderated mediation hypotheses. Bootstrapping with 10,000 resamples was conducted, and bias-corrected 95% confidence intervals were used to determine the significance of indirect effects and conditional effects (Hayes, 2017).

## 3. Results

This section presents the findings in four steps. First, common method bias and multicollinearity were examined to evaluate whether the data met basic statistical assumptions. Second, descriptive statistics and correlation analyses were reported to provide an overview of the study variables and their bivariate associations. Third, mediation analysis was conducted to test whether psychological resilience explained the association between bullying victimization and school adjustment. Finally, moderated mediation analysis and simple slope tests were used to examine whether physical activity moderated the proposed pathways. This sequence allows the findings to move from preliminary data checks to the core hypothesis tests.

### 3.1. Common Method Bias and Multicollinearity Tests

Harman’s single-factor test was used to examine common method bias (Podsakoff et al., 2003). Exploratory factor analysis was conducted in SPSS 26.0 on all items from the Physical Activity Questionnaire for Adolescents, the School Social Behavior Scales, Second Edition, the psychological resilience assessment questionnaire, and the bullying victimization assessment questionnaire. The results showed that, based on the unrotated factor analysis, 14 factors with eigenvalues greater than 1 were extracted. The largest factor explained 25.755% of the variance, which was below the commonly used 40% reference value. Therefore, Harman’s single-factor test did not suggest substantial common method bias in the present study.

A multicollinearity test was also conducted for the independent variable, mediating variable, and moderating variable. The results showed that the tolerance values of all variables ranged from 0.871 to 0.964, exceeding 0.1, and the VIF values ranged from 1.038 to 1.148, all below 10. These findings indicate that there was no serious multicollinearity among the variables.

### 3.2. Descriptive Statistics and Correlation Analysis

The sample consisted of 10,161 male students (53.1%) and 8988 female students (46.9%), with a mean age of 14.68 years.

As shown in Table 1, bullying victimization was negatively associated with psychological resilience (*r* = −0.318, *p* < 0.01) and school adjustment (*r* = −0.465, *p* < 0.01). Psychological resilience was positively associated with school adjustment (*r* = 0.573, *p* < 0.01). Physical activity was positively associated with psychological resilience (*r* = 0.072, *p* < 0.01) and school adjustment (*r* = 0.078, *p* < 0.01). These preliminary correlations were consistent with the proposed theoretical model and provided initial support for the subsequent mediation and moderated mediation analyses.

### 3.3. Relationship Between Bullying Victimization and School Adjustment: Testing the Moderated Mediation Model

To further meet the statistical requirements for mediation analysis, all study variables were standardized. After controlling for gender and age, Model 4 of Hayes’ PROCESS macro for SPSS, version 4.1, was used to examine the mediating effect of psychological resilience. The results (Table 2 and Table 3) showed that, after controlling for gender and age among junior high school students, the direct path coefficient between bullying victimization and school adjustment was significant (*β* = −0.311, *t* = −53.370, *p* < 0.001), with a direct effect of −0.311 and a Bootstrap 95% confidence interval of [−0.329, −0.294]. The path coefficient between bullying victimization and psychological resilience was also significant (*β* = −0.314, *t* = −45.666, *p* < 0.001), as was the path coefficient between psychological resilience and school adjustment (*β* = 0.472, *t* = 81.154, *p* < 0.001). The mediating effect was −0.149, with a Bootstrap 95% confidence interval of [−0.157, −0.140]. These findings indicate that psychological resilience partially mediated the relationship between bullying victimization and school adjustment among junior high school students. Specifically, bullying victimization was negatively associated with school adjustment both directly and indirectly through its association with psychological resilience.

Second, Model 15 of Hayes’ PROCESS macro for SPSS, version 4.1, was used to test the moderated mediation model. After controlling for gender and age, psychological resilience was entered as the mediating variable, and the results are presented in Table 4. After physical activity was included in the model, the interaction between bullying victimization and physical activity significantly predicted school adjustment (β = 0.055, t = 11.014, *p* < 0.001). Similarly, the interaction between psychological resilience and physical activity significantly predicted school adjustment (β = 0.020, t = 4.189, *p* < 0.001). These results indicate that physical activity moderated both the negative association between bullying victimization and school adjustment and the positive association between psychological resilience and school adjustment. However, considering that the large sample size may increase statistical power and lead to statistically significant interaction effects with relatively small effect sizes, the incremental variance explained by the interaction terms was further examined. As shown in Supplementary Table S2, the bullying victimization × physical activity interaction accounted for an additional 0.36% of the variance in school adjustment (ΔR^2^ = 0.0036), whereas the psychological resilience × physical activity interaction accounted for an additional 0.05% of the variance (ΔR^2^ = 0.0005). These findings suggest that although the moderating effects of physical activity were statistically significant, the incremental variance explained by the interaction terms was small. Nevertheless, given that school adjustment is a complex developmental outcome influenced by multiple psychological, behavioral, and contextual factors, even small interaction effects may provide meaningful insights into the conditions under which adolescents maintain better adjustment under peer-related adversity.

To formally examine whether the indirect effect of bullying victimization on school adjustment through psychological resilience varied as a function of physical activity, the index of moderated mediation was calculated using bootstrap procedures. The index of moderated mediation was significant (index = −0.0061, BootSE = 0.0022, 95% CI [−0.0105, −0.0018]), indicating that the indirect effect was significantly moderated by physical activity. The conditional indirect effects at different levels of physical activity are presented in Table 5. Specifically, the conditional indirect effects of bullying victimization on school adjustment through psychological resilience were significant at low, mean, and high levels of physical activity because all bootstrap confidence intervals excluded zero. Although the indirect effects remained negative across all levels of physical activity, their magnitude differed across levels of physical activity, further supporting the moderated mediation hypothesis.

To further verify the moderated mediation model, simple slope analyses were conducted by estimating the effects at one standard deviation below and above the mean of physical activity, representing low and high levels of physical activity, respectively. Specifically, the relationships between bullying victimization and school adjustment, and between psychological resilience and school adjustment, were examined at different levels of physical activity.

**Figure 2:**
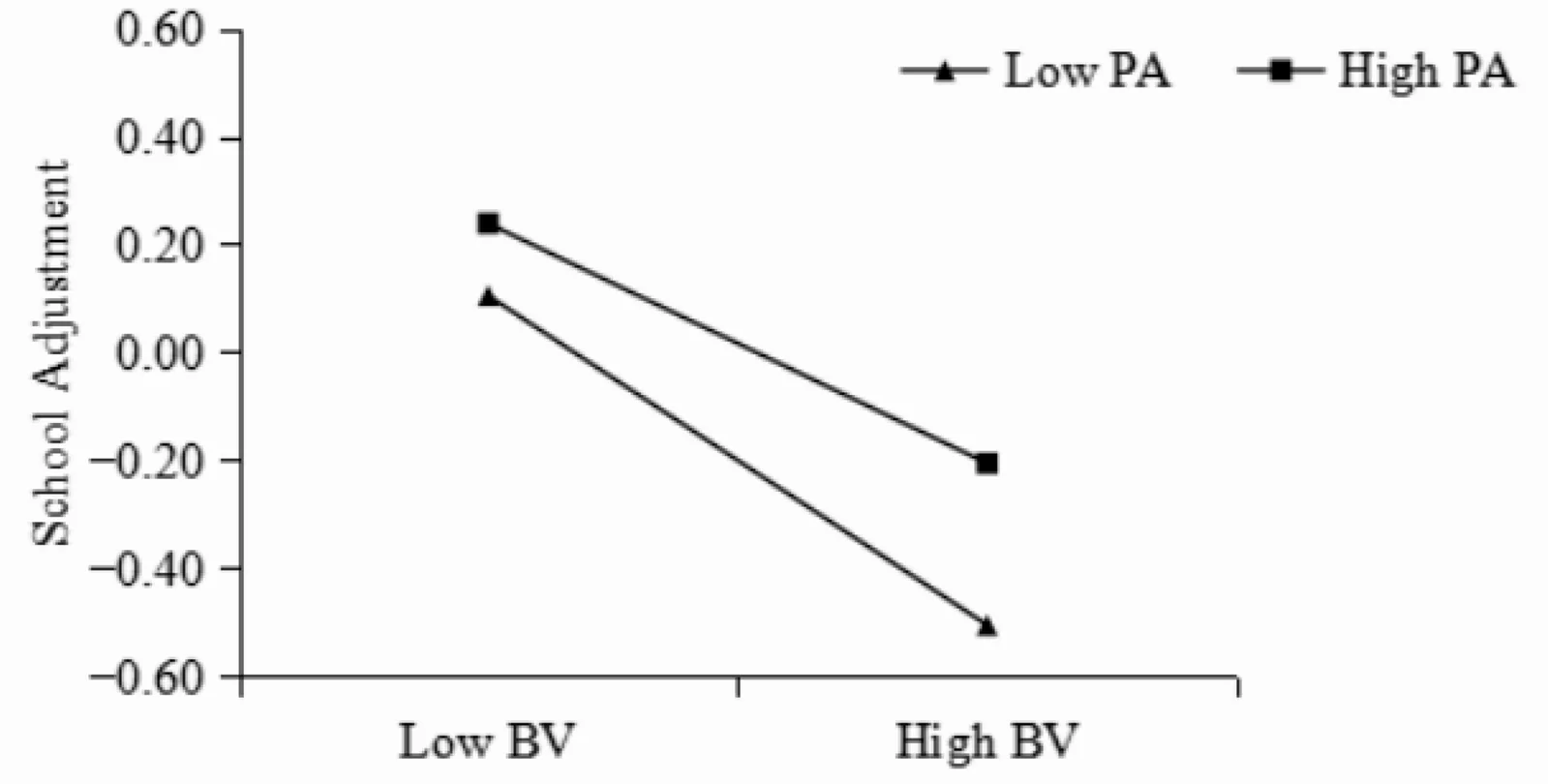
Simple Slope Plot of the Moderating Effect of Physical Activity on the Relationship Between Bullying Victimization and School Adjustment. *Note*. BV = Bullying Victimization; PA = Physical Activity.

As shown in Figure 2, bullying victimization negatively predicted school adjustment at both low and high levels of physical activity, but the association was weaker among students with higher levels of physical activity. This pattern suggests that physical activity may buffer the adverse association between bullying victimization and school adjustment. Detailed simple slope coefficients are presented in Figure 2.

**Figure 3:**
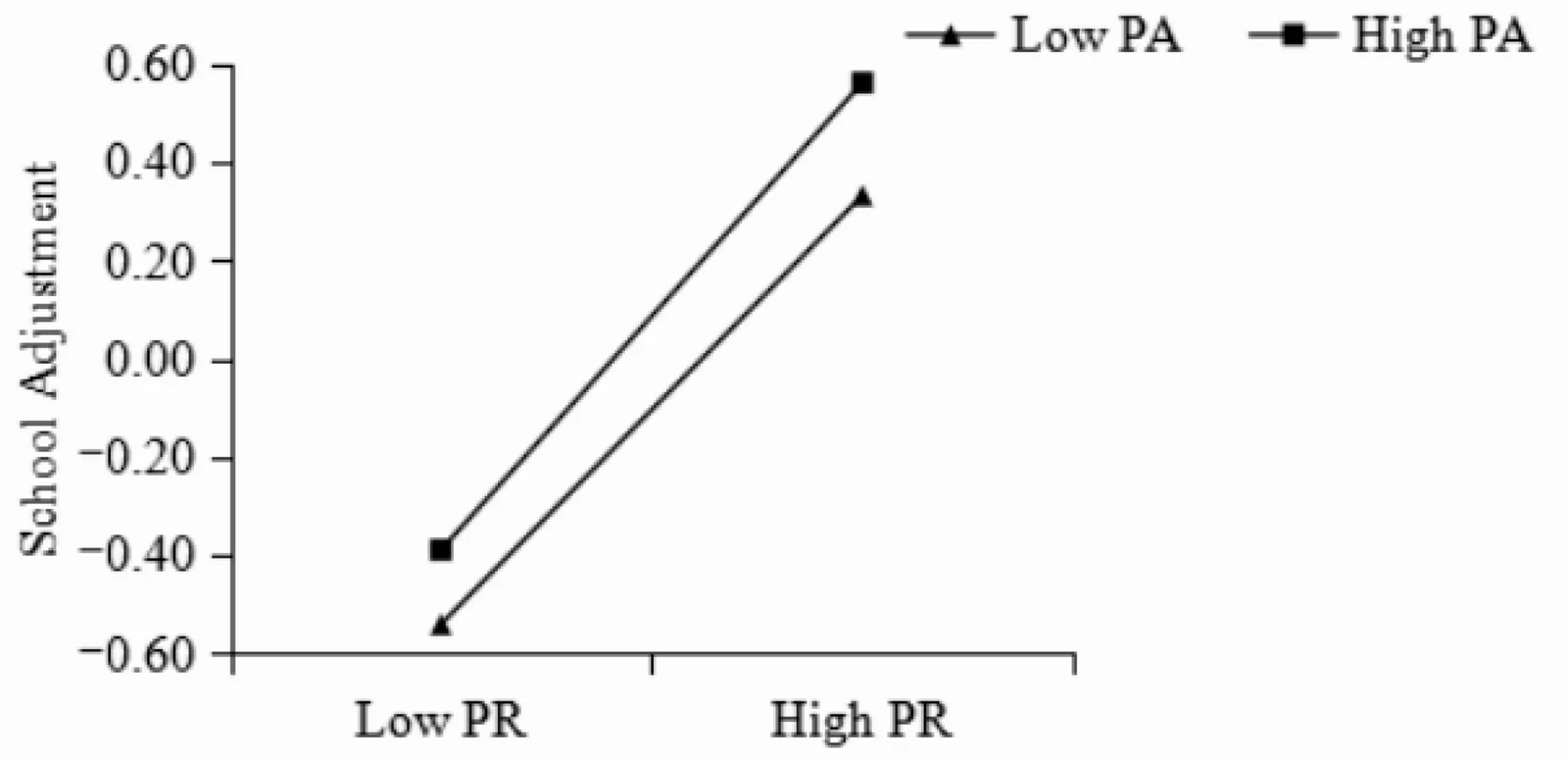
Simple Slope Plot of the Moderating Effect of Physical Activity on the Relationship Between Psychological Resilience and School Adjustment. *Note*. PR = Psychological Resilience; PA = Physical Activity.

In addition, as shown in Figure 3, psychological resilience positively predicted school adjustment at both low and high levels of physical activity, and this association was stronger among students with higher levels of physical activity. This pattern suggests that physical activity may strengthen the beneficial role of psychological resilience in school adjustment. Detailed simple slope coefficients are presented in Figure 3.

## 4. Discussion

Based on stress-coping theory, the risk-protective factor model, and the protective factor-protective factor model, this study systematically examined the relationship between bullying victimization and school adjustment, and further tested the mediating role of psychological resilience and the moderating role of physical activity. The results showed that bullying victimization was significantly and negatively associated with school adjustment among junior high school students. Psychological resilience mediated the relationship between bullying victimization and school adjustment, while physical activity moderated the relationships between bullying victimization and school adjustment and between psychological resilience and school adjustment.

In interpreting these findings, it is important to distinguish between three levels of contribution to the existing literature. First, the finding that bullying victimization negatively predicts school adjustment reinforces prior evidence that peer-related adversity is associated with poorer adolescent adjustment. Second, the mediation result extends previous work by identifying psychological resilience as a pathway linking bullying victimization to school adjustment, rather than focusing only on negative mental health outcomes. Third, the moderation results refine existing literature by showing that physical activity may function not only as a general promotive factor but also as a protective condition under conditions of peer-related adversity. Nevertheless, the incremental variance explained by the interaction terms was relatively small (ΔR^2^ = 0.0036 for bullying victimization × physical activity; ΔR^2^ = 0.0005 for psychological resilience × physical activity), suggesting that physical activity may represent a modest protective resource associated with school adjustment rather than a dominant determinant of school adjustment. Therefore, future interventions should consider physical activity as one component within a broader ecological framework involving psychological, interpersonal, and contextual resources.

### 4.1. Relationship Between Bullying Victimization and School Adjustment Among Junior High School Students

This finding mainly reinforces existing literature by showing that bullying victimization is related not only to emotional distress but also to broader difficulties in school adjustment. It suggests that peer-related adversity may be associated with poorer integration into school life, including lower school engagement, weaker interpersonal relationships, and reduced sense of belonging. This interpretation is generally consistent with previous research (Huang et al., 2023). For junior high school students who experience bullying victimization, being looked down upon, verbally abused, or even physically assaulted by teachers, students, or people outside school may cause psychological and physical harm (Moore et al., 2017). Previous studies have suggested that bullying victimization is not a general form of peer friction, but rather a negative life event characterized by repetition, harm, and power imbalance. Bullying victimization can be regarded as a long-term interpersonal psychological stressor that is associated with poorer emotional states and adjustment functioning (Palamarchuk & Vaillancourt, 2022). According to stress-coping theory, stress is not equivalent to the objective event itself; rather, it is a psychological response formed after individuals conduct primary and secondary appraisals of an event during their interaction with the environment. When individuals appraise a situation as threatening, harmful, or challenging, and perceive their existing resources as insufficient to cope with it, they are more likely to experience maladjustment (Lazarus & Folkman, 1984; Folkman, 2020). Repeated bullying victimization may lead students to appraise peer interactions and the school environment as threatening, thereby weakening their sense of safety, belonging, and self-worth. Existing systematic reviews and meta-analyses have shown that bullying victimization is closely associated with multiple mental health problems among children and adolescents, especially increased risks of internalizing problems such as anxiety and depression (Moore et al., 2017). Related studies have also found that bullying victimization may affect adolescents’ social anxiety through shame and self-esteem, suggesting that bullying victimization may impair individuals’ self-evaluation and induce negative emotional responses (Wu et al., 2021). At the level of school adjustment, studies have shown that bullying victimization reduces adolescents’ sense of school belonging and academic engagement, and further affects their academic achievement (L. Li et al., 2020). Thus, students who experience bullying victimization may not only exhibit negative emotional experiences such as anxiety, fear, shame, and helplessness, but may also encounter adjustment difficulties in learning engagement, school connectedness, and peer interaction. When students are repeatedly harmed by peers but find it difficult to obtain effective coping resources, their perceived safety and sense of control in the school environment may further decline. As a result, they may be more likely to display avoidance, vigilance, insufficient learning engagement, and interpersonal withdrawal, which may be associated with poorer school adjustment.

### 4.2. Mediating Role of Psychological Resilience

The mediating pattern extends previous research by positioning psychological resilience as a potential explanatory pathway underlying the association between bullying victimization and school adjustment, rather than merely as a general protective correlate. This suggests that peer-related adversity may impair school adjustment partly by weakening adolescents’ internal coping resources. This interpretation is consistent with previous findings on the association between bullying victimization and psychological resilience (Deng et al., 2023). Bullying victimization is a persistent, repetitive, and threatening interpersonal stress event. For junior high school students who are in a critical stage of physical and psychological development, long-term exposure to peer aggression or exclusion may keep them in a continuous state of tension, defensiveness, and insecurity, and may gradually lead to negative cognitions about the school environment and peer relationships. According to stress-coping theory, individuals’ adjustment outcomes when facing stressful events depend not only on the stressful events themselves, but also on their cognitive appraisal, psychological resources, and coping processes (Lazarus & Folkman, 1984). When junior high school students repeatedly experience bullying victimization, their perception of threat in the external environment may be continuously reinforced, thereby weakening their emotional regulation, self-support, and positive coping abilities. This is not conducive to the maintenance and functioning of psychological resilience. Therefore, bullying victimization may represent an important risk-related experience associated with reduced psychological resources and lower levels of psychological resilience. In addition, this study found that psychological resilience significantly and positively predicted school adjustment among junior high school students, which is also consistent with previous research (Wu et al., 2024). This result indicates that students with higher levels of psychological resilience usually have stronger emotional regulation abilities, problem-solving abilities, and positive coping tendencies when facing academic pressure, peer conflict, and challenges in school life. Therefore, they are more likely to maintain good classroom participation, interpersonal relationships, and a sense of school belonging. Previous studies have indicated that psychological resilience can help adolescents maintain better psychological regulation when experiencing stressful life events and further promote their school adjustment (Y. Zhang et al., 2019). Thus, psychological resilience is not only an important internal protective resource, but also a psychological foundation for junior high school students to cope with school-related stress and maintain positive adjustment. These findings suggest that the association between bullying victimization and school adjustment may involve psychological resilience as an indirect pathway. On the one hand, bullying victimization may directly undermine students’ sense of safety and belonging in the school environment, causing them to show withdrawal or adjustment difficulties in learning engagement, peer interaction, and school participation. On the other hand, bullying victimization may also reduce psychological resilience, leaving students with insufficient psychological resources and positive coping abilities when facing subsequent stress, thereby further lowering their level of school adjustment. Overall, bullying victimization was associated with poorer school adjustment, and this association may involve psychological resilience as an indirect pathway.

### 4.3. Moderating Role of Physical Activity

#### 4.3.1. Moderating Role of Physical Activity in the Relationship Between Bullying Victimization and School Adjustment Among Junior High School Students

This moderating pattern indicates that physical activity is not only associated with better school adjustment but may also correspond to a weaker negative association between bullying victimization and school adjustment. This finding refines previous studies that mainly treated physical activity as a direct predictor of adjustment, showing instead that physical activity may also function as a behavioral protective condition in the risk pathway from bullying victimization to school adjustment. This finding is consistent with the risk-protective factor model (Fergus & Zimmerman, 2005), which suggests that when individuals are exposed to risk contexts, positive behavioral activities and supportive experiences may buffer the negative effects of risk factors on adjustment outcomes. Physical activity is usually characterized by regularity, interaction, and collectivity, and can provide students with relatively stable opportunities for school participation and positive peer interaction contexts (Eime et al., 2013). Through participation in physical activity, students may obtain more positive emotional experiences through cooperation, competition, rule compliance, and team interaction. These experiences may help improve peer relationships, strengthen school connectedness, and enhance their adjustment within the school environment (H. Chen et al., 2024). Previous studies have shown that physical activity is closely associated with children’s and adolescents’ mental health, social functioning, and positive developmental outcomes (Biddle & Asare, 2011; Lubans et al., 2016). Other studies have also found that physical activity is significantly and positively correlated with school adjustment among junior high school students, indicating that physical activity may be an important factor in promoting students’ school adjustment (Wu et al., 2024). In addition, physical activity may reduce the damage caused by bullying victimization to school adjustment by increasing students’ positive participation experiences at school. For students who experience bullying victimization, such experiences may make them perceive the school environment as unsafe or unfriendly, thereby affecting their learning engagement, peer interaction, and sense of school belonging. In contrast, higher levels of physical activity may provide students with more experiences of success, opportunities for peer cooperation, and positive feedback, potentially reducing the extent to which their overall school experience is characterized by negative experiences associated with bullying victimization.

Existing research has also found close associations among physical activity, bullying victimization, interpersonal relationship distress, and emotion management among junior high school students, suggesting that physical activity may alleviate the adverse effects of bullying-related experiences on students’ adjustment by improving their interpersonal interactions and school life experiences (Q. Zhang & Deng, 2024). Therefore, within the association between bullying victimization and school adjustment, higher levels of physical activity may correspond to weaker negative associations, potentially through greater school participation, improved peer interaction, and more positive school experiences. The simple slope analysis in this study further supported this explanation. Although bullying victimization negatively predicted school adjustment at both low and high levels of physical activity, this negative effect was relatively weaker at high levels of physical activity. This suggests that physical activity can, to some extent, weaken the negative effect of bullying victimization on school adjustment, enabling students to maintain relatively better functioning in classroom participation, peer relationships, sense of school belonging, and overall adjustment to school life.

#### 4.3.2. Moderating Role of Physical Activity in the Relationship Between Psychological Resilience and School Adjustment Among Junior High School Students

This pattern further refines previous research by showing that physical activity may strengthen a protective pathway, namely the positive association between psychological resilience and school adjustment. It is consistent with the protective factor-protective factor model, which suggests that different protective factors do not function in isolation, but may jointly promote positive adjustment outcomes through interactive or synergistic effects (Fergus & Zimmerman, 2005; Zimmerman, 2013). Psychological resilience is an important internal protective factor, whereas physical activity is an external and intervenable behavioral protective factor. These two protective factors may interact in the process of promoting school adjustment among junior high school students. The results of this study further indicated that higher levels of physical activity strengthened the positive association between psychological resilience and school adjustment.

The simple slope analysis further showed that the positive predictive effect of psychological resilience on school adjustment in the high physical activity group was 0.039 higher than that in the low physical activity group. Although the increase was only 0.039, this does not diminish the value of physical activity. Instead, this finding suggests that the role of physical activity in the present study may be more likely to amplify the protective effect of psychological resilience rather than replace its mechanism of action. Specifically, physical activity may, to some extent, strengthen the statistical association between psychological resilience and school adjustment. Therefore, its moderating effect appears as a relatively limited but directionally stable enhancement. This pattern is consistent with the synergistic relationship between protective factors, characterized by “strengthening rather than replacement” (Zimmerman et al., 2013). At the same time, this moderating effect may also be related to the characteristics of school adjustment itself. As a multidimensional adjustment outcome, school adjustment is usually not determined by a single factor. Previous studies have operationalized adolescents’ school adjustment in terms of emotional engagement, school integration problems, and perceived academic performance, and have found that teacher support, peer support, psychological resilience, and positive emotions are all closely associated with school adjustment (Azpiazu Izaguirre et al., 2024). Therefore, the moderating effect of a behavioral protective factor on school adjustment is more likely to be manifested as marginal enhancement on the basis of an existing positive relationship, rather than as a substantial reshaping of the relational structure between variables. In addition, psychological resilience and physical activity may overlap to some extent in their functions of improving emotional states, enhancing self-regulation, and increasing adjustment resources. This means that the added value of physical activity in relation to psychological resilience may be reflected more as complementary reinforcement rather than as a large additional effect (Biddle & Asare, 2011; Lubans et al., 2016). In summary, the findings of this study not only support the positive significance of physical activity for school adjustment, but also further indicate that physical activity can, to some extent, strengthen the protective effect of psychological resilience on school adjustment.

### 4.4. Limitations and Future Directions

Several limitations should be noted. First, this study used a cross-sectional design, so the findings should be interpreted with some caution when discussing causal relationships among bullying victimization, psychological resilience, physical activity, and school adjustment. Although the proposed moderated mediation model was developed based on theory and previous empirical findings, future longitudinal studies could further clarify the temporal relationships among these variables.

Second, the data were collected through self-report questionnaires. Although this method is widely used in adolescent psychological and educational research, it may be influenced by participants’ subjective perceptions, recall bias, and social desirability bias. Moreover, because bullying victimization, psychological resilience, physical activity, and school adjustment were assessed using self-report measures at the same time point, potential common method bias cannot be completely ruled out. Although Harman’s single-factor test did not indicate substantial common method bias, this diagnostic does not definitively exclude its presence. Future studies may combine self-reports with teacher, parent, and peer reports or administrative and objective data, and may collect predictor and outcome variables at different time points, to reduce potential common method bias and provide a more comprehensive understanding of students’ school adjustment and bullying experiences.

Third, this study focused on junior high school students from Shandong Province, China, and some bullying victimization items reflected the Chinese school context, such as being looked down upon by teachers or people outside school. Although these items may help capture locally relevant experiences, they may also limit the comparability of the findings with studies using more standardized international bullying measures. Therefore, future research could further examine whether the findings can be generalized to students from other regions, school stages, or cultural contexts.

Finally, this study mainly examined the roles of psychological resilience and physical activity. Because the present study was based on de-identified secondary data, some potentially important contextual covariates, such as socioeconomic status, school climate, class size, and school-level identifiers, were not available. Therefore, the present analyses could not directly control for these contextual factors or model school-level clustering effects. Because school-level identifiers were unavailable in the database, potential clustering effects among students within schools could not be further examined using multilevel models or cluster-adjusted estimation approaches. Consequently, failure to account for possible within-school dependence may have resulted in underestimated standard errors and overly narrow confidence intervals, thereby potentially overstating the apparent precision and statistical significance of the estimated associations. The reported *p* values and confidence intervals should therefore be interpreted with appropriate caution. Future studies could include additional individual, family, classroom, and school-level factors and retain school-level identifiers to permit the use of multilevel or cluster-adjusted analyses. In addition, physical activity was assessed using a self-reported measure referring to activity during the previous week, which may not fully capture adolescents’ habitual physical activity patterns and may be influenced by recall bias and social desirability bias. Future studies could combine self-report measures with objective assessments, such as accelerometers, to provide a more comprehensive evaluation of adolescents’ physical activity levels.

## 5. Conclusions and Practical Implications

Bullying victimization significantly and negatively predicted school adjustment among junior high school students, and psychological resilience mediated the relationship between bullying victimization and school adjustment. Physical activity moderated both the relationship between bullying victimization and school adjustment and the relationship between psychological resilience and school adjustment.

These findings have several practical implications. For policymakers, anti-bullying policies should not only focus on reducing bullying incidents but also on strengthening school-based protective resources. Schools may consider integrating resilience-building activities, counseling support, and peer-support programs into routine student development services. For practitioners, physical activity may be used as a feasible and low-cost intervention resource to promote positive school experiences, peer interaction, and emotional regulation among adolescents who have experienced bullying victimization. However, physical activity should not be viewed as a substitute for anti-bullying intervention; rather, it should be incorporated as part of a broader school adjustment support system.

## Figures and Tables

**Table 1:** Descriptive Statistics and Correlation Analysis of the Variables.

	M ± SD	1	2	3	4	5	6
1. Gender	0.53 ± 0.499	1					
2. Age	14.68 ± 1.078	0.013	1				
3. Bullying victimization	4.31 ± 2.516	0.084 **	0.059 **	1			
4. Psychological resilience	20.64 ± 4.952	−0.042 **	−0.062 **	−0.318 **	1		
5. Physical activity	2.262 ± 0.681	0.146 **	0.033 **	0.144 **	0.072 **	1	
6. School adjustment	234.01 ± 39.402	−0.081 **	−0.054 **	−0.465 **	0.573 **	0.078 **	1

*Note*. Quantitative variables are presented as mean and standard deviation (SD), and categorical variables are presented as n and percentage (%). ** *p* < 0.01, two-tailed.

**Table 2:** Regression Analysis Results of the Mediation Model.

Regression Equation		Overall Model Fit Indices			Significance of Regression Coefficients	
Outcome Variable	Predictor Variable	*R*	*R* ^2^	*F*	*β*	*t*
School adjustment	Bullying victimization	0.467	0.219	1784.520	−0.460	−71.583 ***
	Age				−0.024	−4.125 ***
	Gender				−0.084	−6.569 ***
Psychological resilience	Bullying victimization	0.321	0.103	734.091	−0.314	−45.666 ***
	Age				−0.040	−6.268 ***
	Gender				−0.030	−2.158 *
School adjustment	Bullying victimization	0.647	0.419	3445.236	−0.311	−53.370 ***
	Psychological resilience				0.472	81.154 ***
	Age				−0.006	−1.105
	Gender				−0.070	−6.349 ***

*Note*. Gender was dummy-coded. * *p* < 0.05; *** *p* < 0.001.

**Table 3:** Bootstrap Test of the Mediating Effect.

	Effect Value	Bootstrap 95%CI		Proportion of Relative Associations
		Boot LLCI	Boot ULCI	
Total effect	−0.460	−0.472	−0.447	100%
Direct effect	−0.311	−0.329	−0.294	67.609%
Indirect effect	−0.149	−0.157	−0.140	32.391%

*Note*. 95%confidence interval estimated using the bias-corrected percentile bootstrap method, respectively. Direct effect: bullying victimization → school adjustment; indirect effect: bullying victimization → psychological resilience → school adjustment.

**Table 4:** Regression Analysis Results of the Moderated Mediation Model.

Regression Equation		Overall Model Fit Indices			Significance of Regression Coefficients	
Outcome Variable	Predictor Variable	*R*	*R* ^2^	*F*	*β*	*t*
School adjustment	Bullying victimization	0.657	0.432	2075.583	−0.347	−56.860 ***
	Psychological resilience				0.457	78.497 ***
	Physical activity				0.095	16.981 ***
	Age				−0.008	−1.579
	Gender				−0.094	−8.474 ***
	Int_1				0.055	11.014 ***
	Int_2				0.020	4.189 ***
Psychological resilience	Bullying victimization	0.321	0.103	734.091	−0.314	−45.666 ***
	Age				−0.040	−6.268 ***
	Gender				−0.030	−2.158 *

*Note*. Int_1 = Bullying Victimization × Physical Activity; Int_2 = Psychological Resilience × Physical Activity. Gender was dummy-coded. * *p* < 0.05; *** *p* < 0.001.

**Table 5:** Index of Moderated Mediation and Conditional Indirect Effects of Bullying Victimization on School Adjustment Through Psychological Resilience at Different Levels of Physical Activity.

Analysis	Physical Activity Level	Index/Effect	BootSE	BootLLCI	BootULCI
Index of moderated mediation	—	−0.0061	0.0022	−0.0105	−0.0018
Conditional indirect effect	Low (−1 SD)	−0.1373	0.0047	−0.1469	−0.1283
	Mean	−0.1435	0.0042	−0.1518	−0.1355
	High (+1 SD)	−0.1496	0.0047	−0.1590	−0.1406

*Note.* The index of moderated mediation and conditional indirect effects were estimated using bias-corrected bootstrap procedures with 10,000 resamples. BootLLCI and BootULCI represent the lower and upper limits of the 95% bootstrap confidence interval, respectively. Low and high physical activity levels represent values one standard deviation below and above the mean.

## Data Availability

The data supporting the findings of this study are available from the Adolescent Health Theme Database, DOI: 10.12213/11.A0031.202107.209.V1.0.

## Supplementary Materials

The following supporting information can be downloaded at: https://www.mdpi.com/article/10.3390/bs16091590/s1, Table S1: Measurement Properties and Confirmatory Factor Analysis Results of the Main Psychological Measures; Table S2: Incremental Variance Explained by Interaction Terms in the Moderated Mediation Model.
