# Supplementary material for: The Impact of Bullying Victimization on School Adjustment: A Moderated Mediation Model
Source: Behav Sci (Basel). 2026 Sep 7;16(9):1590. doi: 10.3390/bs16091590 (PMC13603305; doi:10.3390/bs16091590)
Supplement: Supplementary file 1 [file behavsci-16-01590-s001.zip › behavsci-4462423-supplementary.pdf]

Supplementary file

Confirmatory factor analyses (CFAs) were conducted for bullying victimization, psychological resilience, and school adjustment using Mplus 8.3. Because the indicators were measured on ordinal Likert-type scales, the weighted least squares mean- and variance-adjusted estimator (WLSMV) was used. Standardized factor loadings, internal consistency coefficients, and global model-fit indices are presented in Supplementary Table S1.

For school adjustment, two theoretically plausible measurement models were examined. The first was a single-factor comparison model in which all 64 items loaded on one general factor. The second was a correlated two-factor model comprising Social Competence (Items 1-32) and Antisocial Behavior (Items 33-64). The single-factor model was examined as a comparison model because the source database provides an overall composite score based on all 64 items; it was not assumed to be the preferred measurement structure. As shown in Supplementary Table S1, the correlated two-factor model demonstrated better approximate fit than the single-factor model and was therefore considered a more appropriate representation of the internal measurement structure of the SSBS-2 items.

Importantly, the primary moderated-mediation analyses did not use factor scores derived from the single-factor CFA model. Instead, they used the observed composite index calculated according to the prespecified scoring instructions of the source database. Accordingly, the CFA results support two correlated but distinguishable dimensions, whereas the composite score was retained as a database-prespecified operational index of overall school adjustment and was not interpreted as evidence that the 64 items formed a strictly unidimensional latent scale.

The one-factor model of psychological resilience was specified using all six items prespecified in the source database. These items constituted all psychological-resilience items available for the present secondary analysis, and no items were selected or removed by the present authors. The database documentation described the six items as being adapted from the RSCA but did not designate them as belonging to a specific RSCA subscale or as constituting a separately validated short form. The one-factor model showed satisfactory approximate fit, as reported in Supplementary Table S1. The three-item bullying-victimization model was just-identified; therefore, its global fit indices were not informative and were not interpreted as evidence of perfect fit.

Supplementary Table S1. Measurement Properties and Confirmatory Factor Analysis Results of the Main Psychological Measures

Panel A. Standardized Factor Loadings and Internal Consistency

Measure	Number of items	Range of standardized factor loadings	*Cronbach's α*

Bullying victimization	3	0.849—0.907	0.817
Psychological resilience	6	0.733—0.901	0.885
School-adjustment composite	64	0.525—0.870	0.910

Panel B. Global Model-Fit Indices

Construct and model	χ^2	*df*	*RMSEA*	*CFI*	*TLI*	*SRMR*
Bullying victimization: one-factor model	—	0	—	—	—	—
Psychological resilience: one-factor model	883.602	9	0.071	0.964	0.941	0.069
School adjustment: single-factor comparison model	223491.046	1952	0.077	0.909	0.905	0.078
School adjustment: correlated two-factor model	113496.781	1951	0.055	0.954	0.952	0.056

Note. RMSEA = root mean square error of approximation; CFI = comparative fit index; TLI = Tucker-Lewis index; SRMR = standardized root mean square residual; WLSMV = weighted least squares mean- and variance-adjusted estimator. The correlated two-factor school-adjustment model comprised Social Competence and Antisocial Behavior. The three-item bullying-victimization model was just-identified (df = 0); therefore, its global model-fit indices were not informative and were not interpreted as evidence of perfect fit. For school adjustment, the standardized factor-loading range in Panel A corresponds to the single-factor comparison model, whereas the reliability coefficient refers to the database-prespecified 64-item observed composite.

Supplementary Table S2. Incremental Variance Explained by Interaction Terms in the Moderated Mediation Model

Interaction term	ΔR^2	*F* change	*p*
Bullying Victimization × Physical activity	0.0036	121.314	<0.001
Psychological resilience × Physical	0.0005	17.543	<0.001

activity

Note. ΔR^2 represents the additional variance in school adjustment explained by each interaction term after entering the main effects, covariates, and other predictors included in the moderated mediation model.
